# “Why Do I Need to Come Out if Straight People Don’t Have To?” Divergent Perspectives on the Necessity of Self-Disclosure Among Bisexual Women

**DOI:** 10.3389/fsoc.2021.665627

**Published:** 2021-10-11

**Authors:** Zuziwe Khuzwayo

**Affiliations:** Department of Sociology, University of Witwatersrand, Johannesburg, South Africa

**Keywords:** bisexuality, coming out (or disclosure), narrative life history, global south, queer theory

## Abstract

*Coming out* has historically been an important yet often very challenging process for LGBTQI + individuals to no longer conceal their sexual and/or gender identity. For those who identify as bisexual, the process of coming out has proven especially complicated. In the general knowledge field of sexual identity, bisexuality continues to be a misunderstood, under-researched sexual identity, and from that negative stigmas and discrimination (even within LGBTQI + spaces) have contributed to bisexuals not *coming out* even within the LGBTQI + community. However, the significance and necessity of *coming out* itself has come to be questioned, particularly by younger LGBTQI + people. From a PhD study conducted in Johannesburg with 23 self-identifying bisexual women, this paper critically considers the different perspectives on *coming out* of bisexual women. Using a narrative life-history approach through interviews with a sample of eight participants from the study, this paper looks at how bisexual women understand the significance of *coming out* and how this process has different meanings for different age groups. Findings show that there are vastly divergent perspectives, with some participants believing it remains essential, while others argue that the fluidity of their identities no longer requires the same sort of disclosure.

## Introduction

In the Global North and in some former colonized countries, the disclosure and recognition of one’s sexual identity for non-heterosexual individuals has historically been significant in shaping their identity (Brownfield et al., 2018). This process usually occurs through *coming out* (CO) whereby and individual claims their non-heterosexuality, politically or personally. Historically for bisexual individuals, the process of CO has been challenging as bisexuality continues to be under-researched and misunderstood, which has led to negative stigmas towards this identity and thus contributing to an apprehension to disclose (Barker and Landridge, 2008).

Studies on non-heterosexual studies have existed as early as the 20th century (Mead, 1970) and over the years there has been a growing body of research focused on non-heterosexual identities, which have challenged the dominant heterosexual understanding of sexual identities (Butler, 2006). This growth in research combined with activism from the Lesbian, Gay, Bisexual, Transgender, Intersex and other identities (LGBTQI+) movement has shifted attitudes about LGBTQI + individuals and their rights globally (Flores and Park, 2018). In some parts of the African continent this shifting of attitudes is met with challenging ideas that homosexuality is “unAfrican” and is a Western ideology and another form of colonialism (Epprecht, 2013). In the South African context, there is a history of activism of non-heterosexual rights, but even in these spaces bisexual identity does not always receive the same acknowledgement as lesbian and gay rights (Chan-Sam, 1994; Stobie, 2004; Lynch and Maree, 2013).

The emergence of queer studies in the 1980s and 1990s, which focuses on the socially constructed theories on gender, sexual practices, and sexual orientation (Carnes, 2019), has allowed for a fluid perspective when discussing sexual identity and a defiance against the heterosexual binary understanding of sexual identity (Butler, 2006). Transnational queer theory allows for individuals to be able to describe and define their sexual identity taking into consideration other issues of race, class, age, and geography that have not always featured in White Global North understandings of sexual identity (Currier and Migraine-George, 2016). For bisexual individuals living in the Global South, this has been significant as firstly it has allowed for issues such as race, age, gender, class, and the impact of colonialism to be discussed when trying to understand non-heteronormative sexualities. Second, it has brought into discussion different ways of describing and performing multiple identities that challenge heteronormativity, which is privileged in Global North and former colonial societies (Martin and Kazyak, 2009).

During this period of the 1970s and 1980s a neoliberal ideology was being adopted by states globally. Economically this ideology argues for a reduction of state interventions in economic and social activities and the deregulation of labour and financial markets (Clare, 2017). This ideology influenced feminist movements and brought about a post-feminist movement. This ideology argued that gender equality between the “traditional sexes” had been reached with the legal victories gained by the previous women’s movements and that women were now “empowered” and therefore they should seek their new “freedom” in traditional understandings of women’s roles and abandon feminist ideologies (McRobbie, 2004; Dosekun, 2015). This was exemplified in popular culture in music, tv, and film where women were asserting their sexual freedom, and in the workplace where women were occupying positions that were traditionally not held by them (Nash and Grant, 2015). Critical to both examples and the pillar of post-feminism is consumer culture and the idea of “individual choice” in relation to sexual freedom that women now have acquired, rendering feminist ideologies as outdated (Dosekun, 2015; Banet-Weiser and Portwood- Stacer, 2017).

In South Africa, the LGBTQI + movement has early roots in Johannesburg as the 1970s and 1980s homosexual-sub-culture was prominent in the city and from that, organisations such as the Lesbian and Gays Against Oppression (LAGO) and the Gay and Lesbian Organisation of Witwatersrand (GLOW) were formed to fight against the discrimination of LGBTQI + individuals (Cameron, 1994). Johannesburg continues to be an important space for LGBTQI + individuals to raise political awareness and socialize with other LGBTQI + individuals. There are safe spaces in the city for LGBTQI + individuals to be able to disclose their sexual identity without the threat of violence (Bagnol et al., 2010). In the South African context, the influence of neoliberalism in LGBTQI + spaces also occurred in the new democratic dispensation where the African National Congress (ANC) came into power and with it a neoliberal ideology (Oswin, 2007). This influenced the LGBTQI + movement in that it moved away from the radical feminist politics of freedom and equality, but rather towards the advancement of individual self-hood, an assimilation and social acceptance of heteronormative institutions (e.g., marriage), and a failure to recognize the intersectional identities (race, class, and gender) that affect LGBTQI + individuals in the country (Oswin, 2007). A reason for this is because South Africa had enshrined in its democratic constitution the legal recognition of LGBTQI + individuals, which was assumed to be the end of the fight against injustice and a discarding of feminist ideologies (Oswin, 2007).

## History of Bisexuality

In Global North studies the documented history of bisexuality can be traced back to the ancient period of the Greeks and Romans, where bisexuality was part of the culture and men and women were known to engage in opposite-sex relationships as well as same-sex relationships (Cantarella, 2002). According to MacDowall (2009), p. 4), bisexuality in the 19th century was defined as “forms of life that exhibit physical characteristics of both sexes.” This created the idea of bisexuality being about individuals with both sexual organs. Sexual identities were not prescribed at the time and it was only in the early 20th century where concepts of homosexuality and heterosexuality were developed and discussed as studies on individual’s sexual desires started to grow. Even though studies started to grow on homosexuality, bisexuality was never recognised and studied as a sexual identity even though individuals were engaging in bisexual relationships (Rust, 2002). Freud in his work acknowledged bisexuality as a sexual identity but viewed it as a transitory state and from which an individual will mature into either heterosexual or homosexual (Carey, 2005). Significant research by Alfred Kinsey with the introduction of the Kinsey scale has emphasized the fluidity of sexual identity, and the idea of bisexuality being the “middle ground” of multiple sexual identities (Hemmings, 2002). Critical work by Diamond (2008) has taken it a step further and looked at bisexual women and recognizing their sexual fluidity in terms of their love and desires. Both of this work is significant, but unfortunately not enough has been done looking at women in the Global South and how their bisexual identity is shaped by other factors such as race, class, age, and space.

Colonialism not only brought economic changes, but it also reinforced the existing patriarchal order between the sexes, hierarchy between the races, and most critical was that it brought a heteronormative belief on sexual orientation through Christian ideology (Amadiume, 1987). The belief of heterosexuality being the only sexual identity has persisted on the continent as the significant influence of Christianity and other religions which believe in heterosexuality as the only sexual identity and the belief of non-heterosexuality as “UnAfrican” and a new form of colonialism (Wahab, 2016). There has been some progress on the continent with the enshrinement of rights of non-heteronormative individuals in some countries and research showing how non-heteronormative sexual identities have existed on the continent before and during colonialism (Epprecht, 2004); however, ideas of heteronormativity on sexual identity on the continent persist. It must be noted that the acceptance has mainly been of homosexual individuals and not enough on other sexual minority groups and from that a hetero/homo binary has been created when discussing sexual identities (Flores and Park, 2018).

With its refusal to fit into the binary understandings of sexual identity and being the “middle ground,” bisexuality has faced de-legitimation as a sexual identity through a concept defined by Yoshino (2000) as “bisexual erasure” which shows the multiple ways in which bisexuality in society is erased and not recognised as a sexual identity in both heterosexual and homosexual spaces. Second, bisexual identity is constantly questioned as a valid identity as the influence of post-feminism has brought a discourse of female sexual empowerment that encourages women to “experiment” or be “curious” in their sexual practices with other women, but still through a heteronormative male gaze rendering a bisexual identity as “a phase” and not a legitimate sexual identity (Grant, 2018). Along with this, sexual identity studies continue to be mono-normative: understanding of sexual identity in binary terms where homosexuality and heterosexuality are rendered the only valid forms of identity, and it is assumed that a person is attracted to another person of either the same or a different (“opposite”) gender (Hayfield et al., 2014). In recent years, there has been some international research that has explored this topic (e.g., Pallotta-Chiarolli, 2014; Boyer and Galupo, 2015; Lahti 2015), but far less on the African continent.

Both of the above factors have meant that bisexuality continues to be an invisible and “silenced” sexual identity, which has meant that bisexual individuals have not always been willing to disclose their sexual identity as they fear the stigma and misunderstandings that come with it and, in some instances, this has caused mental health issues for bisexuals (Knous, 2006; Lynch and Maree, 2013; Hayfield et al., 2014). From the African continent critiques have been made on the act of CO and how it is not indigenous to the continent and a Western act for non-heterosexuals (Wa Tushabe, 2017; Miguel, 2021). This will be discussed further in the paper when looking at CO where the origin of the act is questioned.

## Bisexuality in South Africa

Historically in South Africa the LGBTQI + resistance can be traced to the beginnings of colonialism in 1652 with the arrival of the Dutch that included the introduction of Christianity to the indigenous people of the country (de Gruchy and de Gruchy, 2005). There were many attempts to evangelize the indigenous San & Khoi people, but this was sporadic and was not successful in the beginning until the introduction of missionary schools in the 19th century that were able to instil heterosexuality as the only sexual identity. Homosexual activities continued and individuals were not legally persecuted, but their activities were deemed socially unacceptable (Thompson, 1990).

It was the Immorality Act of 1957 that, among other things, prohibited same-sex relations that brought together gay organizations that mobilised against this legislation and restriction of movement, but these organisations were mainly made up of White men (Cameron, 1994). Historically Black LGBTQI + individuals were not able to legally create or access public spaces in which to discuss or explore their concerns related to their sexual identities. It was during the 1980s that LGBTQI + organizations outside of White urban areas and within anti-Apartheid structures were formed (Cameron, 1994). Organizations such as the Lesbians and Gays Against Oppression (LAGO), the Organisation of Lesbian and Gay Activists (OLGA), and the Gay and Lesbian Organisation of Witwatersrand (GLOW) were established and advocated for the rights of all LGBTQI + individuals regardless of race (Cameron, 1994).

Bisexuality like other sexual labels has historically been defined from Global North countries, and these definitions have been used globally without taking into consideration local contexts outside of these spaces (Milani and Lazar, 2017). In South Africa, the word “stabane” has had multiple meanings. During the gold rush in the late 1800s the word was used by Zulu men in the mines, and it was associated with banditry and “sodomy” as men were stealing from each other or sleeping with each other in the hostels (Marwick, 1918). Another use of the word has been in a derogatory manner to refer to an individual who has both male and female sexual organs and they were considered to be “bisexual.” Today it is used to refer to LGBTQI + individuals in an offensive manner (Swarr, 2009). As mentioned earlier the LGBTQI + movement in South Africa historically acknowledged bisexuality but did not give bisexuality the same attention as compared to lesbians and gay individuals and the prejudice and bigotry they face (Chan-Sam, 1994). For bisexuals there was an added layer of prejudice as bisexuals faced judgment within the LGBTQI + movement as bisexuality in some corners was not considered a valid identity (Chan-Sam, 1994). This then made bisexuals at the time unwilling to self-identity as bisexuals and rather say that they are sexually fluid or homosexual (Chan-Sam, 1994). The challenges of understanding and legitimizing bisexuality persist in present-day South Africa, and this makes it difficult for bisexuals to be open about their sexual identity.

## Understanding Queer Theory

Sexual identities, ideas, and practices stemming from Global North countries do not consider how sexual identities outside of the Global North are influenced by different economic and social contexts, especially for women (Salo et al., 2010). An example of this occurs in India where “kothi” is a term used to describe individuals who are on the spectrum of gender identity and do not fit into Global North ideas of transgender individuals. This has led to this group being socially and economically marginalized as they are not seen as being “respectable,” and they do not fit into disseminated Global North ideas of sexual identity, especially those who present as female (Dutta, 2013; Reddy, 2005). Historically ethnographic studies of the early 20th century looked at how multiple factors influenced sexual identities (Mead, 1970), but it was during the 1980s and 1990s that queer theory developed a perspective on understanding non-heterosexual identities as socially constructed and fluid in their performativity, and not a fixed identity (Butler, 1997). Examples of this are sexual practices (polygamy), ways of embodiment (sadomasochist) and desires (multi-partner sex) that have nothing to do with the sex or gender of the object choice but significantly shape an individual’s sexual identity. Queer theory has evolved to include intersectional identities such as race, class, age, and geography and how these factors influence the sexual identity of an induvial (Dutta, 2013; Valocchi, 2005). This is critical for bisexuality as it complicates the binary understanding of sexual identity (“middle ground”) and rather it argues for a contextual and fluid understanding of sexual identities. Scholars have associated queer theory with bisexuality because it transgresses the idea of single-sex attraction and rather a fluid understanding of sexual identity (Jagose, 2009).

In South Africa like in other global contexts the word “queer” was historically used in a negative manner to refer to non-heterosexual individuals (Matebeni and Msibi, 2015). It has only been since the democratic dispensation of 1994 that the word has referred to LGBTQI + individuals in a positive manner and has been used by scholars and activists to look at how different factors such as race, class, age, and space shape the lives of LGBTQI + individuals living in South Africa and the challenges they face in relation to their sexual identity (Livermon, 2012; Tucker, 2009). This is critical for bisexual women in the country as multiple factors have influenced how they sexually identify and what acts they perform to claim their identity. Critical to this paper is the practice of CO and how local contexts have shaped bisexual women’s acceptance and refusal of this act for multiple reasons.

## Coming Out

CO has been viewed as a critical act in an individual’s identity development. It signifies individuals claiming their non-heterosexuality in their public and private lives (Craig and McInroy, 2014). Historically, in Global North countries, it has been a political act in gaining legal recognition and equality and it has been a social act in an individual choosing to live authentically in their sexual lives (Brownfield et al., 2018). This act has been used by some former colonised countries such as South Africa where LGBTQI + individuals of different race, class, and age have used the act of CO combined with activism to gain legal recognition and rights for themselves (Gevisser, 1994). This act has also been found to be positive for some bisexual women as it allows them to gain social acceptance and integrate into their communities once they disclose their sexual identity (Baiocco et al., 2020).

As mentioned earlier the misunderstanding and under-researching of bisexuality has invisibilised the sexual identity and so therefore CO is sometimes accompanied with a stigma and biphobia, which makes bisexuals reluctant to disclose (Wandrey et al., 2015; Knous, 2006; Corrigan and Matthews, 2003). For bisexual women the stigma of bisexuality is exemplified by it being viewed as a “phase,” not a legitimate identity, and being promiscuous which has historically made it difficult or unwilling of bisexuals to disclose their sexual identity (Flanders and Hatfield, 2014; Scherrer et al., 2015). Over the past decade scholars and young non-heterosexuals have argued that CO is a way of conforming to heteronormativity by seeking heterosexual acceptance, which creates a constant asserting of an individual’s sexual identity which some non-heterosexuals refuse to engage with (Craig and McInroy, 2014).

The act of CO continues to allow for safe spaces for LGBTQI + individuals to share their experience, but in certain contexts issues of race and gender have influenced whether individuals choose to disclose their sexual identity. The influence of a neoliberal post-feminist perspective on CO does not take into consideration contextual factors that influence whether an individual will “come out.” In the United States during the HIV/AIDS crisis LGBTQI + individuals of colour were reluctant to come out in fear of rejection when they needed family and community during the crisis (Drucker, 2015). Even though legal rights have been gained for non-heterosexuals in South Africa, harassment and violence continue, especially towards Black lesbian women (Matebeni, 2013). Non-heterosexual Black women living in South Africa have experienced homophobia through physical violence, which in a significant number of cases has resulted in death (Gqola, 2015; Gunkel, 2010). This has made it very difficult for non-heterosexual Black women to *come out* outside of LGBTQI + safe spaces in the fear of possibly facing violence. For bisexual women, the threat of violence is more nuanced as Black bisexual women engaging in same-sex relationships do face the threat of violence as they will be seen as homosexual, but if they engage in relationships that appear to be heterosexual (i.e., man and woman) they will “pass” as heterosexual and so therefore the threat of violence will not occur unless they decide to come out (Wandrey et al., 2015). All the above has put into focus the necessity of CO and finding alternative ways to disclose that will not be life-threatening, limiting in understanding multiple sexual identities and will be dependent on the individual to disclose if they deem necessary in their contexts.

This paper will look at how different bisexual women living in Johannesburg have divergent perspectives on CO, and how some have resisted the idea as a way of challenging the heteronormativity of understanding sexual identity.

## Data Analyses

### Procedures

The data for this article were obtained from a PhD study titled “Bisexuality in Democratic South Africa: Experiences of Women in Johannesburg.” The study focuses on how bisexual women construct their sexual identity looking at multiple factors such as race, class, age, and how different spaces of Johannesburg shape how they perform their sexual identity. The criteria to select participants was that participants had to identify as bisexual women, were 18 years or older, and were currently living in Johannesburg. Initial conversations with participants involved explaining the research and providing examples of questions such as *How would you define bisexual identity? Do you disclose your bisexual identity to your romantic partners?* and *What does coming out mean to you?* in order for them to understand the study and some of the topics that would be discussed during the interview.

The methodology used for this research was a narrative life-history approach. This approach allows for a deeper level of data capturing to occur by gaining deeper insight into different contexts which have shaped the individual’s experiences by relying heavily on individuals’ subjective realities; however, the focus is not on objective facts and accuracy of people’s stories, but instead on the meaning life events have on the participants. From these narratives, meanings can be produced, and substantive theories can be formulated based on interpretation of reality, rather than scientific explanation (Dhunpath, 2009; Bertaux and Kohli, 1984). Through in-depth interviews with the participants, data was gained to be able to understand how participants came to understand their sexual identity from an early age, how race, class, and space has shaped their sexual identity, and for this paper how they understand CO and whether they think it is still a necessary act to perform. Previous studies with LGBTQI + individuals that have used this approach recognize how this approach allows for participants to be reflective and thoughtful when discussing their sexual identity and for their voices to come through in the research (Webb, 2014; Quesada and Vidal-Ortiz, 2015).

To gain participants for the study, multiple sampling methods were used. The first method was through snowball sampling, which allowed me to obtain participants through other research participants by using social networks (friends, family, workplace, organizations, etc.). This sampling method allows access to participants easily who are willing to participate in research (Noy, 2008). Social media was the second tool used in the research and it was significant as it allowed for unasked questions in a less formal atmosphere to be discussed and for marginalized groups to be able to discuss issues in an open public format (Kirkup, 2010). This tool was critical as it allowed access to participants outside of known networks and a broader selection of participants. This type of sampling also allowed for access to participants who feared stigma, prejudice, or marginalization because of their bisexuality and were not easily accessible but wanted to participate in the research. Twitter and Facebook were used, and a brief note was posted on both platforms requesting to interview women over the age of 18 years old who identify as bisexual, who may or may not be “out of the closet” and were willing to participate in the research. The data was collected between 2019 and 2020 and the analysis of the data was done with Atlas. ti software.

Ethical clearance was obtained from the Human Research Ethics Committee (Non-medical) of the University of Witwatersrand. A participant information sheet as well as a participant consent form was handed out to participants for them to understand the study as well as agree in signature to be a part of the study.

### Participants

Using the criteria mentioned earlier, 23 participants were selected for the PhD study, and quotes from 8 of the participants were selected which reinforced the divergent perspectives. The average of all the participants was 28 years old, the predominant race was Black African (65%), half of the participants did not subscribe to any religion (52%), and most of the participants were single (57%). The sociodemographics of the participants is provided in Table 1.

**TABLE 1:** Sociodemographics of participants.

Pseudonym	Age	Race	Profession	Religious affiliation	Relationship status
Kgomotso	27	Black African	Spiritual healer	Christian	In a relationship
Michelle	58	White	Lecturer	Jewish	In a relationship
Beth	34	White	Lecturer	None	Single
Tshepiso	26	Black African	Teacher	Atheist	In a relationship
Nana	25	Black African	Intern Journalist	Muslim	Single
Kopano	22	Black African	Student	None	Single
Jennifer	28	White	Consultant	Christian	In a relationship
Babalwa	24	Black African	Debating Coach	Christian	In a relationship
Dineo	22	Black African	Student	None	Single
Ayanda	25	Black African	Journalist	None	In a relationship
Lindiwe	21	African Asian	Student	Muslim	Single
Karima	29	Black African	Freelance Editor	None	Single
Nomvula	21	Black African	Student	None	Single
Zinzi	29	Black African	Consultant	None	Single
Lauren	45	Coloured	Graphic Designer	None	In a relationship
Kimberly	33	White	Video Journalist	Christian	Single
Amanda	24	Black African	Self-Employed	None	Single
Lerato	21	Black African	Student	Christian	In a relationship
Pumla	26	Black African	Lawyer	Christian	Single
Mpho	35	Black African	Medical Doctor	Jehovah’s Witness	Single
Rachel	35	White	Development Specialist	None	Single
Bokang	27	Black African	Lecturer/PhD Student	None	In a relationship
Rachel	28	White	Tattoo Artist	None	In a relationship

All the participants identified as cis-female bisexuals except for one who identified as gender non-conforming (Tshepiso) and used the pronouns she/they.

## Results

A summary of the results is provided in Table 2.

**TABLE 2:** Summary of results.

Theme	Description	Quote
Agency and authenticity of non-heterosexual identities	CO is important as it allows bisexual women to gain agency over their sexuality and be authentic to themselves and others about their sexual identity	*Acknowledgement and authenticity about my bisexuality. I think to a very large extent, it’s about authenticity, pride and casting off shame and owning my agency. I love who I love, and I will not apologise for that especially as a woman. (Michelle, 58)*
Validity of Bisexuality	CO is important as it allows for bisexual identity to be recognized as valid and legitimate for some participants	*Coming out is important for bisexual individuals because not only are you saying you are not straight, but you are bisexual which is different from being gay or straight. Bisexuality indicates that you are attracted to more than one gender or non-gender conforming individuals. It is important for people to understand that about bisexuality*. (*Beth, 34*)
Refusal to be Othered	CO is not important as it is a conforming to binary understanding of sexual identity and heteronormative understanding of sexuality which must be rejected	*I don’t believe in it and I don’t subscribe to it. It makes me feel othered like I am different not in a good way. (Simphiwe, 21)*

### Agency and Authenticity of Non-heterosexual Identities

Previous research has shown that the act of CO is important for bisexual women as it allows individuals to have choice in whether they self-disclose their sexuality and a way in which they can have their sexual identity accepted firstly by themselves and then by others (Baiocco et al., 2020; Knous, 2006). Out of the 23 participants selected, 10 of the participants highlighted the importance of CO to them and why it was a necessary action to perform. For these participants one of the primary reasons for CO is that it represents a claiming of their sexual identity and being truthful to their bisexual identity. Michelle, a 58-year-old White woman, commented that for her CO is about

*Acknowledgement and authenticity about my bisexuality. I think to a very large extent, it’s about authenticity, pride and casting off shame and owning my agency. I love who I love, and I will not apologise for that especially as a woman. (Michelle, 58)*

Another participant, Kgomotso, a 27-year-old Black African woman, commented that for her CO means

*Speaking the truth about who you are confidently and not quietly because you own it. (Kgomotoso, 27)*

Both Kgomotso’s and Michelle’s statements highlight the idea of how CO is about being authentic to who they are and no longer having shame as a bisexual woman. Second, the statements highlight how CO represents agency in choosing their sexual identity and using their agency to love whomever they desire.

### Validity of Bisexuality

As mentioned earlier, the binary understanding of sexual identity has resulted in the invisibility of sexualities that do not conform to the dualistic framework (Butler, 2006). CO allows for bisexuals to challenge this form of erasure and ensure delegitimization does not occur. For participants CO is an opportunity to make bisexuality more visible. Beth, a 34-year-old White woman, commented:

*Coming out is important for bisexual individuals because not only are you saying you are not straight, but you are bisexual which is different from being gay or straight. Bisexuality indicates that you are attracted to more than one gender or non-gender conforming individuals. It is important for people to understand that about bisexuality. (Beth, 34).*

Michelle commented:

*For me, bisexuality is a very specific and material sexuality. It’s not a lapse. It’s not a half-half. Bisexuality is about the capacity to feel desire for men and for women. (Michelle, 58).*

For these participants CO continues to be relevant as first it signifies individuals disclosing their non-heterosexuality and being truthful to who they are, which remains a challenge. Second and most critically, it signifies the recognition of bisexuality, which moves away from a binary lens of sexual identity and rather a fluid understanding of sexual identities.

### Refusal to Be Othered

The act of CO can be understood as a way in which individuals still have to conform to heterosexual/homosexual understandings of sexual identity and not embracing a fluid understanding of sexual identities (Charania, 2005). This is influenced by a neoliberal sexual politics that argues for an acceptance and assimilation into heteronormativity rather than a rejection of it completely (Grant, 2018). For 13 of the participants interviewed, they did not see the relevance and the need to come out. For these participants they felt that CO still indicates difference of non-heterosexuality in a negative manner, which they did not want to conform to. Simphiwe, a 21-year-old Black African woman, commented:

*I don’t believe in it and I don’t subscribe to it. It makes me feel othered like I am different not in a good way. (Simphiwe, 21)*

Tshepiso, a 26-year-old Black African gender non-conforming individual, commented:

*I hate the construct of coming out. Like I always think it means that we are always fighting for equality. Why do I need to come out if straight people don’t have to? (Tshepiso, 26)*

Another participant, Nana, a 25-year-old Black African woman, felt that CO meant that she had to fit into limited understandings of sexual identity, which ultimately does not fully describe who she is. She commented:

*It is not really important to me. I just am who I am. I feel like coming out breeds the ground for a person to then box and categorise me which I don’t like so I don’t tell people I am not straight. Also it is a very Western understanding of sexuality which I am currently thinking through and do not always agree with. (Nana, 25)*

Kopano, a 22-year-old Black African woman, just like Tshepiso felt that CO was a way of giving power to the idea of heterosexuality being the norm which she did not want. For her CO meant

*Living in your truth and not expecting acceptance from people. Asking to be accepted gives straight people that power and I don’t want that. I will live my life whether you accept me or not it doesn’t change the fact that I am a bisexual woman. (Kopano, 22)*

Lastly Jennifer, a 28-year-old White woman, objects to the action of CO but rather “inviting in” as a way of claiming her sexual identity. She commented:

*I don’t believe in coming out, but rather “inviting in.” Inviting in suggests that I don’t owe you the right to tell you about my queerness, but rather I choose who to tell and when I do tell you I will not be held responsible for doing the work, but if you want to still be a part of my life, I invite you to do the work and accept me and my sexuality. (Jennifer, 28)*

For these participants CO is not only of no importance to them but also a refusal to allow others to have power of their sexual identity on whether it is acceptable or not. The refusal to conform and even a suggestion of a different way of disclosing your sexual identity indicates a refusal to conform to binary sexualities and a refusal to engage in traditional ways of disclosing, which tend to still favour heteronormative understanding of sexualities.

## Discussion

### The Importance of CO in Legitimizing a Misunderstood Sexual Identity

For some of the bisexual women interviewed, CO is an important act. It represents the idea of ownership and acknowledgment of their sexual identity. Historically bisexual individuals were, and in some countries continue to be, a marginalized group who face criminal charges for their sexual identity, and CO is an act in which they use to fight for their legal rights. This is particularly true in South Africa where previously there were discriminatory laws as mentioned earlier. In the new dispensation discriminatory laws have been removed but biphobic beliefs persist, and CO is used to challenge these negative beliefs. It is evident that for the participants over the age of 25 CO is still influential as they grew up during a period where CO was used as a form of activism not only against the Apartheid system but also against stigma and prejudices that have existed against bisexuals in LGBTQI + spaces. CO can still be used as an act to challenge these negative ideas and show how bisexual individuals experience biphobia which is distinct from other forms of homophobia. CO is still relevant for these bisexual women as it represents an opportunity to clarify misconceptions and ideas on bisexuality and legitimize their sexual identity, which is critical for bisexual individuals (Barker and Landridge, 2008).

Participants still feel CO is important as it indicates the recognition of a bisexual identity and for it to be viewed as a valid sexual identity and not have it seen through a binary lens. This then emphasizes the importance of queer theory in how one understands bisexual identity as it allows for multiple sexual identities to be acknowledged and deemed legitimate and, most importantly, shifts away from the binary understanding of sexual identity and towards a fluid understanding. This is evident with the comments from Beth (34) and Michelle (58) who view CO as an important way to claim their sexual identity that is distinct from other non-heterosexual identities.

The significance of the ages of the participants who still view CO as important highlights a difference in generational perspective on CO. For these older bisexual women, they still view CO as important and a way in which to validate their sexual identity as compared to women under the age of 25 who do not see CO as an act to achieve this. This signifies a generational shift in attitudes towards CO and its relevance with younger bisexual women.

As mentioned earlier, CO is important as it allows individuals to gain self-acceptance as well as acceptance from others in their community (Knous, 2006). For the bisexual women interviewed it has allowed them to be honest about their sexual identity and not have negative feelings surrounding their sexual identity. Research has shown bisexual individuals experience higher levels of poor mental health as compared to other non-heterosexuals, and reasons for this are the erasure of a bisexual identity and a lack of support that bisexuals have in their communities (Hayfield et al., 2014; Scherrer et al., 2015). This is evident with Kgomotso (27) and Michelle who view CO as a way for them to acknowledge their sexual identity and no longer have shame in claiming it personally and in public spaces. This act has allowed participants to legitimize their identity and to potentially educate others about a bisexual identity and not have it viewed in a negative manner.

The high levels of violence against same-sex relationships among women in South Africa can be viewed as a method to regulate women’s bodies and force them into heteronormativity (Gqola, 2015). For the bisexual women interviewed, CO allows them an opportunity to have agency over who they choose to have a romantic relationship with, and a way in which to publicly challenge the regulation and marginalizing ideology on sexual identity. This is evident with Michelle who recognizes the importance of CO as a way to have agency over her body and her sexual identity by choosing to love whomever she desires as a bisexual woman.

### Rejection of Heteronormativity

Some of the younger bisexual women interviewed do not agree with the act of CO. First, for these participants CO represents an act of constantly having to validate their sexual identity to heterosexual individuals, which they refuse to participate in. The act of CO can be seen to represent a form of heteronormativity that they challenge. An example of this can be seen with Kopano (22) who views CO as a form of acceptance from heterosexual individuals, which she is not looking for, neither does she want heterosexuals to think they have any influence as to how she chooses to identify as a bisexual woman. This then highlights the significant influence that heteronormativity still has in society, even in marginalized spaces, and how individuals must make a conscious effort to resist even with their sexual identity (Butler, 2006).

Second, some bisexual women rejected the act of CO because it represented the idea of seeking equality as non-heterosexual individuals. CO is an act that is only associated with LGBTQI + individuals and an act that they must perform in order to gain legal rights in certain countries. For the younger participants they reject CO because that means that they always have to be fighting for equality and heterosexuals do not have to perform this act at all in order to be viewed as equal in terms of the law. This is made clear with Tshepiso’s (26) comment where they challenge the act of CO as a way of constantly fighting for equality and not just having equality like straight people. For younger bisexual women interviewed, CO is an outdated act to gain equality that they don’t necessarily seek as it is under heteronormative understandings of sexual identity. The act of not CO can therefore be seen as not only a refusal but also a form of activism in a non-traditional manner where individuals are not engaging in spaces that require them to seek legitimacy, acceptance, or do some form of labour in order to be viewed as equal.

Third, the bisexual women interviewed who do not agree with the act of CO reject the act because it represents a binary understanding of sexual identity, which participants do not necessarily fit into particularly because they identify as bisexuals. CO still has heterosexual/homosexual connotations to it which does not adequately take into consideration the fluidity of multiple sexual identities which do not fit into this binary, and therefore participants reject this act (Craig and McInroy, 2014). This is illustrated in Nana’s (25) comment where CO feels like a categorisation exercise that does not describe her sexual identity, and it makes it very difficult for her as a bisexual woman to engage with.

Lastly, the rejection of CO by the younger bisexual women interviewed indicates how this act does not resonate with bisexual women from the Global South. CO is a Global North idea that does not take into consideration the nuances of how sexual identity is understood in post-colonial countries (Wa Tushabe, 2017). Nana’s comments indicate this as she questions the idea of CO as “a very Western understanding of sexuality,” which is a foreign way of gaining acceptance and legitimacy as a bisexual individual and forces the individual to align with Global North ideas of LGBTQI + individuals, which she is thinking through, and hence she rejects the idea of CO. In South Africa, where race and class still have a significant role in shaping sexual identities, particularly for Black women, the threat of violence because of their sexual identity which does not conform to the heteronormativity is a possibility, hence the unwillingness to engage with an act that could pose a threat to their lives (Matebeni, 2013; Gqola, 2015). This could possibly be a reason why Nana rejects the act of CO as it does not take into consideration her context as a Black bisexual woman and the possible violent threat she faces if she *comes out*.

In this study younger bisexual women on average rejected the idea of CO. This could indicate how younger bisexual women are engaging in different forms of queer activism by refusing to engage in terms and languages that no longer serve who they are or their beliefs on their sexual identity. In all of the above rejections of CO, it is clear that there is a need for a queer theoretical approach which better understands how sexual identity is constructed in Global South contexts, how multiple factors need to be considered when discussing sexual identities, the recognition of the fluidity of bisexuality, and how it does not fit in a binary framework (Currier and Migraine-George, 2016).

### Moving Towards Different Forms of Language and Ideas

The last comment by Jennifer (28) identifies a different way in which individuals can begin, if they choose, to claim their sexual identity publicly on their own terms. The idea of “inviting in” first suggests a choice on whether to perform the act of CO. Bisexual individuals do not have the obligation to “come out” as a way of being authentic in who they are but rather the agency lies with them and whether they choose to invite others in informing them on their sexual identity (Johns, 2020). Second, the idea suggests a selection that an individual makes in choosing who to include in how they identify sexually. This immediately puts the power in the individual’s hands and not necessarily in others (family, friends, colleagues, etc.) to accept who they are.

The idea of “inviting in” does not seek the legitimacy from Global North heteronormative understandings of sexual identity, but rather suggests a journey into understanding who the individual is and how it is they perform their sexual identity (Moore, 2012). Last, the idea does not put the labour on the individual to constantly validate their sexual identity by constantly asserting who they are, but rather shifts that work to those who seek to be in community with the individual and asks these individuals to engage in reciprocal labour in order to be a part of the individual’s community (Johns, 2020). Jennifer indicates this in her comment by making it clear that she does not owe anyone the right to inform them on her sexual identity, but rather she is using her agency to choose who she invites in informing them of her sexual identity.

This action can be viewed as a different type of activism which places power with the individual and does not seek legitimacy from Global North heteronormative understandings of sexual identity which can be limiting, but rather a recognition of fluidity and contextual differences when discussing sexual identity.

The present study contributes to the tension that women who identify as bisexual view the act of CO from a Global South context. For older bisexual women in the study CO is still a relevant act to perform in order to gain acknowledgment and legitimacy of bisexual identity which continues to be questioned as a valid sexual identity. This act is a significant opportunity for bisexual women to claim their sexual identity which may not always occur as assumptions will be made on their sexual identity depending on what type of romantic relationships they are presenting in public, which could present them as either being heterosexual or homosexual. In the South African context, acknowledgement and some social acceptance of LGBTQI + individuals has occurred, and this has informed how younger bisexual women no longer view the act of CO as necessary, but rather they reject the act and are using different methods of acknowledgement and legitimacy in relation their sexual identity. This informs the idea that CO is a Global North act which is not indigenous to Global South contexts in understanding sexual identities, in this case the bisexual identity of women, where multiple factors influence their sexual identity and their refusal to fit into Global North understandings of bisexual identity.

Future research aims to focus on how women who identify as bisexual living in rural areas outside of Johannesburg view CO. In South Africa most of the safe LGBTQI + spaces are in metropolitan urban cities and bisexual women living in rural areas may be reluctant to *come out* as safe LGBTQI + spaces are sparse in those areas. The idea of space (i.e., geography) could have a significant influence on whether bisexual women *come out* and whether they view the act as important.

### Limitations of the Study

The study did have some limitations. The first limitation is the number of women who identified as bisexual was very few even though multiple methods to select participants were used. It could be that some bisexual women were not willing to participate in the research because they feared the stigma and discrimination that they could potentially face. The second limitation was that of the participants willing to participate in the study, most of them came from middle-class backgrounds and not from other classes, and this limited how class plays a role in how bisexual women view CO in Johannesburg. Lastly as mentioned previously the study was conducted in the urban city of Johannesburg and not in the rural areas outside of Johannesburg where bisexual women living in those spaces are affected by different factors which will influence how they view CO.

## Conclusion

This paper has shown the different perspectives on CO and how it varies for bisexual women from the Global South. CO remains an important political act for some of the bisexual women as it represents a way of claiming their sexual identity, while for others, especially those who are younger, it is no longer relevant as they view it as another way in which heteronormativity and Global North understandings of sexual identities persist, which they reject. For the bisexual women interviewed, the idea of legitimacy is important as bisexuality continues to be a marginalized and discriminated sexual identity, but there are differences in how they choose to validate that sexual identity especially in contexts where race, class, and age have influenced how individuals shape their sexual identity. Queer theory and activism offer a way in which bisexuality can be viewed and understood, and “inviting in” offers a new way in which non-heterosexuals can begin to claim legitimacy over their own sexual identity on their own terms without the burden of having to constantly do the labour of seeking acceptance and legitimacy.

## Data Availability

The original contributions presented in the study are included in the article/Supplementary Material, further inquiries can be directed to the corresponding author.
